# Molecular Epidemiology and Genetic Diversity of Zika Virus from Field-Caught Mosquitoes in Various Regions of Thailand

**DOI:** 10.3390/pathogens8010030

**Published:** 2019-03-06

**Authors:** Atchara Phumee, Rome Buathong, Rungfar Boonserm, Proawpilart Intayot, Nucharat Aungsananta, Akanitt Jittmittraphap, Yutthana Joyjinda, Supaporn Wacharapluesadee, Padet Siriyasatien

**Affiliations:** 1 Thai Red Cross Emerging Infectious Diseases-Health Science Centre, World Health Organization Collaborating Centre for Research and Training on Viral Zoonoses, Chulalongkorn Hospital, Faculty of Medicine, Chulalongkorn University, Bangkok 10330, Thailand; amphumee@gmail.com (A.P.); yutthana.jjd@gmail.com (Y.J.); spwa@hotmail.com (S.W.); 2 Vector Biology and Vector Borne Disease Research Unit, Department of Parasitology, Faculty of Medicine, Chulalongkorn University, Bangkok 10330, Thailand; sky_rung123@hotmail.com; 3 Bureau of Epidemiology, Department of Disease Control, Ministry of Public Health, Nonthaburi 11000, Thailand; romebua@hotmail.com; 4 Medical Science Program, Faculty of Medicine, Chulalongkorn University, Bangkok 10330, Thailand; khunproaw@gmail.com; 5 Public Health Center 22 Wat Pak Bor, Health Department, Bangkok Metropolitan Administration, Bangkok 10250, Thailand; thephonee@gmail.com; 6 Department of Microbiology and Immunology, Faculty of Tropical Medicine, Mahidol University, Bangkok 10400, Thailand; akanitt@hotmail.com

**Keywords:** Zika virus, mosquitoes, molecular epidemiology, genetic diversity, Thailand

## Abstract

Zika virus (ZIKV) infection is an emerging and re-emerging arbovirus disease that is transmitted to humans through the bite of infected mosquitoes. ZIKV infections were first described in Thailand in 1954 from the sera of indigenous residents and several travelers returning from Thailand in 2014. However, reported cases in Thailand have been increasing since 2015 and 2016, and epidemiological information about the vectors of ZIKV is unclear. We investigated the molecular epidemiology and genetic diversity of ZIKV from mosquitoes collected from different geographic regions experiencing ZIKV outbreaks in Thailand. Polymerase chain reaction was used to amplify the non-structural protein (*NS5*) gene of ZIKV, which was then sequenced. A total of 1026 mosquito samples (626 females, 367 males, and 33 larvae) were collected from active ZIKV patients’ houses. ZIKV was detected in 79 samples (7.7%), including *Aedes aegypti* (2.24% female, 1.27% male, and 0.19% larvae), *Culex quinquefasciatus* (1.85% female, 1.66% male, and 0.29% larvae), and *Armigeres subalbatus* (0.1% female and 0.1% male), whereas no ZIKV was detected in *Aedes albopictus*. Phylogenetic analysis of the 79 positive samples were classified into two clades: Those closely related to a previous report in Thailand, and those related to ZIKV found in the Americas. This is the first report of the detection of ZIKV in *Ae. aegypti*, *Cx. quinquefasciatus*, and *Ar. subalbatus* mosquitoes, and genetic variations of ZIKV in the mosquitoes collected from several geographic regions of Thailand were examined. Detection of ZIKV in male and larval mosquitoes suggests that vertical transmission of ZIKV occurred in these mosquito species. This study provides a more in-depth understanding of the patterns and epidemiologic data of ZIKV in Thailand; the data could be used for future development of more effective prevention and control strategies of ZIKV in Thailand.

## 1. Introduction

The Zika virus (ZIKV) is a mosquito-borne flavivirus, closely related to dengue, Japanese encephalitis, West Nile, and yellow fever viruses [1]. ZIKV is transmitted primarily by *Aedes* mosquitoes. ZIKV is classified into African and Asian lineages [2,3]. In 1947, ZIKV was first found in a forest in Uganda in rhesus macaque monkeys. ZIKV was first isolated from an *Aedes africanus* mosquito collected at the same site [4] and documented in humans in 1952 from Uganda and the United Republic of Tanzania [5]. In 1966, ZIKV was first isolated in Asia from *Ae. aegypti* mosquitoes collected in Malaysia [6]. In Thailand, ZIKV infections were first described in 1954 in the sera of autochthonous residents using neutralizing antibodies against ZIKV [7]. There are some reports of ZIKV cases among Canadian [8], German [9], and Japanese [10] travelers returning from Thailand in 2014. Buathong et al. [11] described seven autochthonous acute ZIKV infections cases from the Ratchaburi, Phetchabun, Sisaket, and Lamphun provinces, confirmed by molecular or serological testing. The phylogenetic tree showed that these ZIKV belonged to the Asian lineage. During January to November 2016, a total of 686 Zika infection cases were reported in Thailand by the Ministry of Public Health (MoPH) [12]. The MoPH reported the first two indigenous cases of ZIKV-related microcephaly in Asia since September 2016 [13]. Although human ZIKV infection cases had been reported, data are lacking on ZIKV in mosquito vectors in Thailand. Many studies reported that *Aedes* mosquitoes such as *Ae. africanus*, *Ae. aegypti*, *Ae. apicocoargenteus*, *Ae. furcifer, Ae. vitattus*, and *Ae. luteocephalus* are the principle vectors of ZIKV in Africa [14,15,16,17]. In Southeastern Senegal, several mosquito species are probable potential vectors for ZIKV transmission, including *Ae. aegypti*, *Ae. africanus*, *Ae. furcifer*, *Ae. luteocephalus*, *Ae. vittatus*, *Ae. taylori*, *Ae. dalzieli*, *Ae. hirsutus*, *Ae. metallicus*, *Ae. unilinaetus*, *Culex perfuscus*, *Mansonia uniformis*, and *Anopheles coustani* [18]. In Southeast Asia, potential vectors of ZIKV transmission include *Ae. aegypti* [19] and *Ae. albopictus* [20]. Our previous studies found that *Ae. aegypti* and *Armigeres sulalbatus* are naturally infected with ZIKV in Thailand [21]. All the reports indicated that ZIKV infection is emerging, re-emerging, and increasing in many different areas, in addition to the discovery of many mosquito species naturally infected with ZIKV. Therefore, finding naturally ZIKV infected mosquitoes is essential for understanding the epidemiology of ZIKV infection. In this study, we investigated the potential vectors, molecular epidemiology, and genetic diversity of ZIKV in mosquitoes from several affected regions in Thailand by using Hemi-nested real-time polymerase chain reaction (hn-RT-PCR) of the non-structural protein (*NS5*) gene. Information obtained from the study can be applied to develop effective control strategies for ZIKV infection in Thailand. The nucleotide sequence data also provide fundamental information for the application of molecular techniques for future development.

## 2. Results

### 2.1. ZIKV Infection in Mosquitoes

**Figure 1:**
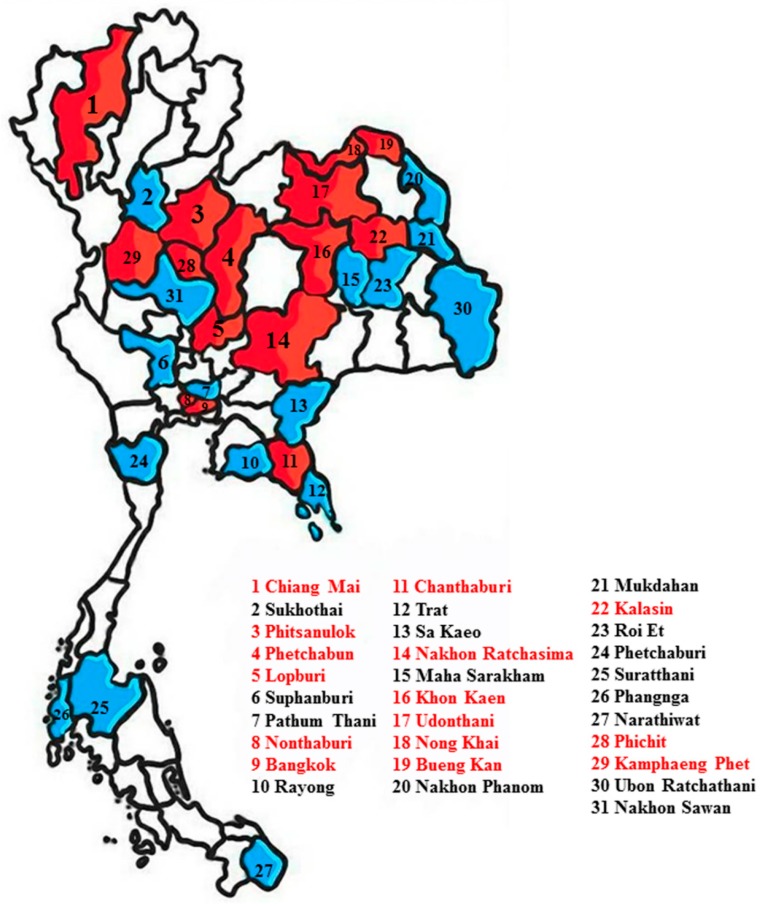
Map of Thailand showing locations of the sample-collection sites in the 31 provinces of 6 regions in affected areas. Red denotes the collection locations of positive Zika virus (ZIKV) in mosquitoes and blue denotes negative ZIKV in mosquitoes.

**Figure 2:**
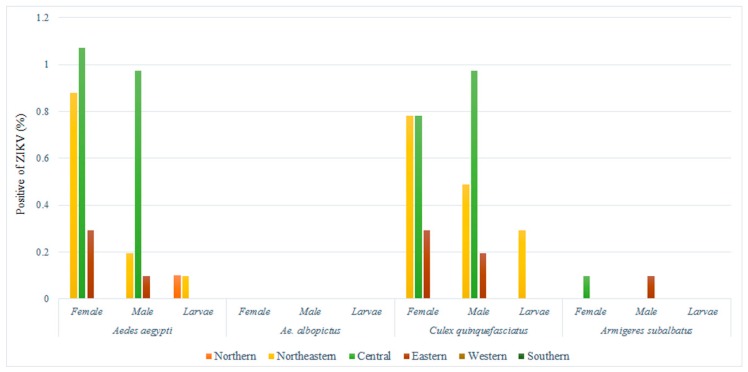
Distribution of ZIKV infection for each area of endemicity.

A total of 1026 mosquito samples were collected in the 31 provinces of six regions in affected areas in 2016, including 626 females, 367 males, and 33 larvae. Positive samples were found in 15 provinces, of which most were found in the central region (Figure 1). ZIKV RNA was detected in 7.7% (79/1026) of mosquitoes, of which 4.19% positive samples were found in female, 3.03% in male, and 0.48% in larval mosquitoes. Mosquitoes infected with ZIKV were found in *Ae. aegypti* (2.24% female, 1.27% male, and 0.19% larva), *Cx. quinquefasciatus* (1.85% female, 1.66% male, and 0.29% larva) and *Ar. subalbatus* (0.1% female and 0.1% male) (Figure 2), whereas we were unable to detect ZIKV RNA in *Ae. albopictus*. The ZIKV sequences showed 98–100% sequence identity with the partial *NS5* genes of ZIKV available in the GenBank database. For the first time, we detected ZIKV RNA in *Cx. quinquefasciatus* among female, male, and larvae in Thailand. We think that *Ae. aegypti*, *Cx. quinquefasciatus*, and *Ar. subalbatus* should be considered vectors of ZIKV infection in Thailand.

### 2.2. Sequencing and Phylogenetic Analysis

**Figure 3:**
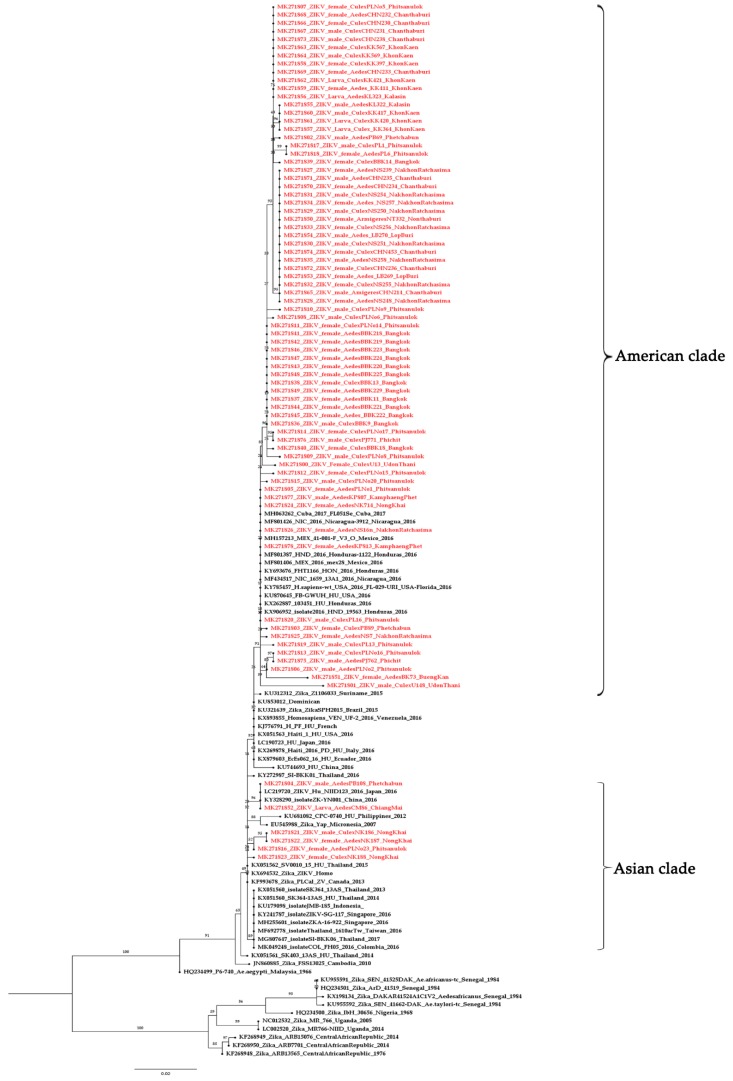
Phylogenetic tree of ZIKV mosquitoes constructed from partial *NS5* sequences from all region of Thailand. The maximum likelihood was constructed with IQ-TREE by using the maximum-likelihood method with 1000 ultrafast bootstrap replicates. The best-fit model of substitution was found using the auto function on the IQ-TREE web server. The sequences from this study are indicated with a red color.

**Figure 4:**
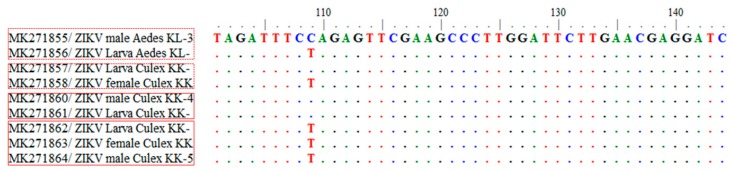
Comparison between sequences of ZIKV obtained from different mosquito stages.

The 79 sequences, when compared with the GenBank database, were similar to ZIKV and showed the clade to be within the Asian lineage. The nucleotide sequences of *NS5* of ZIKV were submitted to the GenBank database, accession numbers MK271800–MK271878. We report the genetic variations in ZIKV from mosquitoes collected from several geographic regions. The ZIKV from mosquitoes in this study can be classified into two clades: The Asian clades from Chiang Mai, Nong Khai, Phitsanulok, and Phetchabun, closely related to a previous report in Thailand [22,23]; and the American clade related to ZIKV found in the Americas (Figure 3). The intraspecific variation analysis between the Asian and the American clade showed 1–6% variation. For the interspecific variation in this study, the Asian lineage showed 15–19% variation from the African lineage. Sequences of ZIKV obtained from adults and larvae of same mosquito species were compared. Some adults and larvae were 100% identical and some showed differences in ZIKV sequences between adults and larvae of the same mosquito species (Figure 4).

## 3. Discussion

To the best of our knowledge, few studies have investigated the genetic diversity of ZIKV in *Ae. aegypti*, *Cx. quinquefasciatus*, *Ae. albopictus*, and *Ar. subalbatus*, which are commonly found in anthropized tropical and subtropical areas, especially in Thailand. Since 2016, reported cases of ZIKV in Thailand have increased. With no vaccine or specific treatment, the major approach to prevention and control of ZIKV infection is vector control [24]. A previous report suggested that virus transmission depends on the mosquito species, geographic location, and virus type [4]. Thus, study of the molecular epidemiology and genetic diversity of ZIKV from mosquitoes in different geographic regions in Thailand is necessary. In this study, we conducted a molecular epidemiologic and phylogenetic analysis of ZIKV in six regions of Thailand in 2016. The results provide an overview of the genetic diversity of ZIKV in epidemic regions. Of the collected mosquitoes, 7.7% of female, male, and larval mosquitoes were positive for ZIKV in *Ae. aegypti*, *Cx. quinquefasciatus,* and in both female and male *Ar. subalbatus*; whereas ZIKV RNA was not detected *Ae. albopictus*. However, some *Ae. aegypti* and *Cx. quinquefasciatus* contained blood meals, and the positive ZIKV RNA from these samples may have caused an overestimation of the virus infection rate in mosquitoes if the mosquitoes fed on a person’s blood that contained ZIKV. The literature shows that the ZIKV isolated from genus *Culex*, *Anopheles* or *Mansonia* suggests the potential role of these mosquitoes as secondary vectors in ZIKV transmission [25]. However, other findings showed that ZIKV may have a wider range of vectors through both vector competence assay and field studies with natural ZIKV infection [22]. For example, *Ae. aegypti* and *Ae. albopictus* were capable of transmitting ZIKV in a vector competence assay and through natural infection [20,26,27,28]. *An. coustani* [18] and *An. gambiae* [29] were naturally infected with ZIKV and are probable vectors in Senegal. Several reports showed that *Cx. quinquefasciatus* is a potential ZIKV transmission vector and that ZIKV can replicate in *Cx. quinquefasciatus.* [30,31].

Our report is the first in Thailand that detected ZIKV RNA in different developmental stages (larva and male and female adults) of *Cx. quinquefasciatus.* Our results support that transovarian transmission of ZIKV occurs in *Ae. aegypti* and *Cx. quinquefasciatus* mosquitoes in the field. In contrast, *Cx. torrentium* and *Cx. molestus* from Germany [32], as well as *Cx. sitiens* and *Cx. annulirostris* from Australia [33,34], were not susceptible to ZIKV. *Cx. pipiens* from Italy [35], Tunisia [36], Germany [32], and the U.S. [37] were not infected when exposed to ZIKV through an artificial blood meal. Our results indicate that *Cx. quinquefasciatus* could be another potential vector for ZIKV transmission in Thailand. The reason we did not find ZIKV in *Ae. albopictus* is that *Ae. albopictus* displays exophagic and exophilic behavior. The mosquitoes prefer to feed outside houses, and after feeding, they rest outside houses, especially in the tree crop such palm trees, rubber plantation. However, in this study, we collected mosquitoes in and around houses. This why the number of *Ae. albopictus* collected in this study was low, so additional studies on *Ae. albopictus* and ZIKV in Thailand should be completed in the future. Further studies are required to investigate additional geographical areas using a larger sample size of mosquitoes.

We investigated the phylogenetic relationships of 79 of the sequences of *NS5*-ZIKV in mosquitoes using the maximum likelihood (ML) mapping method with 1000 replicates. *NS5* is essential for the replication of the flaviviral RNA genome [38,39,40]. Two major lineages (African and Asian) of ZIKV were identified using *NS5*-RT-PCR [41]. The 79 positive samples showed that the ZIKV in this study belongs to the Asian lineage (Asian clade and American clade). The ZIKV found in mosquitoes collected from the same area were classified into the same clade, such as Bangkok (MK271836–271849). The Asian clade was detected in both *Aedes* and *Culex* in the adult and larval stages. However, in some areas such as Phitsanulok, ZIKV was found both Asian and American clade in *Aedes* mosquitoes. We were interested in exploring differences in ZIKV from mosquitoes that were classified into the two clades. Our phylogenetic analysis revealed numerous sequence variations (15–19%) of ZIKV mosquitoes between the African and Asian lineages, as well as among different regions (1–6%) within the Asian lineage. *NS5* could be used for classification and determination of the genetic variations in ZIKV, and our report provides fundamental data for further epidemiological studies of ZIKV in Thailand. A report by Lanciotti et al. (2016) [42] demonstrated that ZIKV obtained from Guatemala and Puerto Rico are all within the Asian lineage and most closely related to ZIKV isolation from Brazil (2015) and French Polynesia (2013). They assumed that it is possible that Asian lineage viruses may have been evolving and spreading geographically throughout Asia and the Pacific Islands since at least 1966 [43] as well as having probably occurred through the travel or living of people in endemic areas. This can explain why we found American-clade ZIKV in our study. However, little information is available about the molecular epidemiology, evolution, and ecology of ZIKV. Our findings therefore suggest that it is likely that the genetic diversity of ZIKV is underestimated due to the limited sequence data currently available for humans in Thailand. Therefore, extensive surveys and more precise studies of ZIKV infection in patients covering more areas and larger sample sizes must be performed in order to understand geographic location and virus type interaction. In this study, we detected ZIKV RNA in whole mosquitoes, so the role of *Cx. quinquefasciatus* and *Ar. subalbatus* for ZIKV transmission should be investigated. ZIKV transmission in expectorated saliva of *Cx. quinquefasciatus* and *Ar. subalbatus* should be studied to obtaining more accurate information. The vector control strategies for ZIKV outbreaks should focus on other mosquito species, and the larval control measures should focus on the breeding sites of *Cx. quinquefasciatus* and *Ar. subalbatus*.

## 4. Materials and Methods

### 4.1. Ethics Statement

The study was approved by the animal research ethics committee of Chulalongkorn University Animal Care and Use Protocol (CU-ACUP), Faculty of Medicine, Chulalongkorn University, Bangkok, Thailand (COA No. 023/2560).

### 4.2. Sample Collection

A total of 1026 larval or adult mosquitoes were collected from active ZIKV infected patients’ homes in 2016. The collections were conducted in different geographical regions of Thailand, including the northern (Chiang Mai), north-eastern (Bueng Kan, Kalasin, Khon Kaen, Nakhon Ratchasima, Mukdahan, Maha Sarakham, Udon Thani, Nong Khai, Roi Et, Ubon Ratchathani, and Nakhon Phanom), central (Bangkok, Kamphaeng Phet, Lop Buri, Nonthaburi, Phetchabun, Phitsanulok, Phichit, Nakhon Sawan, Sukhothai, Pathum Thani, and Suphan Buri), eastern (Chanthaburi, Rayong, Trat, and Sa Kaeo), western (Phetchaburi), and southern (Narathiwat, Phangnga and Surat Thani) regions. Mosquitoes were collected in and around houses of confirmed ZIKV infected patients using mosquito aspirators, and larvae were collected in and around houses of ZIKV infected patients using a large-mouth plastic dropper or a plastic cup. These samples were differentiated according to their sex and species based on morphological identification, separated into pools of 10 individuals, and kept in liquid nitrogen and transferred to the laboratory of Vector Biology and Vector Borne Disease Research Unit, Department of Parasitology, Faculty of Medicine, Chulalongkorn University for viral detection. Larvae were reared into adults and used for species identification and ZIKV detection. Individual mosquitoes were used for ZIKV detection.

### 4.3. Viral RNA Extraction

All mosquito samples were ground in 1× phosphate buffered saline (PBS) and centrifuged at 11,000× *g* for 10 min. The supernatant was used for viral RNA extraction by using a viral RNA extraction kit, Invisorb^®^ Spin Virus RNA Mini kit (STRATEC molecular GmbH, Berlin, Germany) following the manufacturer’s instructions. RNA concentration and purity were quantified by a Nano Drop 2000c spectrophotometer (Thermo Fisher Scientific, MA, USA). The extracted RNA samples were used for ZIKV detection immediately and the rest of samples were stored at −80 °C.

### 4.4. ZIKV RNA Detection

The RNA samples extracted from the mosquitoes were amplified for detecting ZIKV at its *NS5* gene by hemi-nested RT-PCR (hn-RT-PCR), following a process modified from Moureau et al. [44]. The hn-RT-PCR amplification reaction was set up in a final volume of 25 μL using the Superscript III one-step RT-PCR kit (Invitrogen, Grand Island, NY, USA). The nested PCR was performed with 2 μL from the first reaction using 1 unit of *Taq* DNA polymerase (Thermo Fisher Scientific, MA, USA). The PCR products were analyzed via 2% agarose gel electrophoresis, stained with ethidium bromide, and visualized with Quantity One Quantification Analysis Software Version 4.5.2 (Gel DocEQ System; Bio-Rad, Hercules, CA, USA). We constructed the lower band of synthesized positive control plasmid as a positive control to prevent the contamination of the samples.

### 4.5. Gel Purification and Sequencing

Positive PCR products were recovered from the gel and purified using an Agarose Gel DNA Purification Kit: Invisorb^®^ Fragment CleanUp (STRATEC molecular GmbH, Berlin, Germany) following the manufacturer’s instructions. The purified DNA was sent for direct DNA sequencing to 1st Base Laboratories. (Axil Scientific, Singapore). Nucleotide sequences were analyzed by comparison with the GenBank database using a BLAST program.

### 4.6. Phylogenetic Tree Construction

The sequences were aligned using BioEdit Sequence Alignment Editor Version 7.2.5 [45]. The phylogenetic trees were constructed using the maximum-likelihood method with IQ-TREE on the IQ-TREE web server (http://iqtree.cibiv.univie.ac.at/) with 1000 ultrafast bootstrap replicates. The best-fit model of substitution was found using the auto function on the IQ-TREE web server [46]. The phylogenetic tree was finally viewed and edited with FigTree v1.4.4 software (http://tree.bio.ed.ac.uk/software/figtree/). All sequences in this study were analyzed with 40 reference strains of Asian lineage (GenBank accession no. KX051560, MK049248, MG807647, MH255601, MF692778, KU179098, KY241787, LC219720, KY328290, HQ234499, KX051560, KX051561, KY272987, KX051562, MH157213, MF801406, MH063262, MF801426, MF434517, MF801387, KX906952, KY785457, KY693676, KX262887, KU681082, KU853012, KX879603, KU870645, KJ776791, KU744693, LC190723, KX051563, KX269878, KX893855, JN860885, EU545988, KF993678, KU312312, KX694532, and KU321639) and 10 reference strains from the African lineage (GenBank accession no. NC012532, LC002520, HQ234501, HQ234500, KF268949, KF268950, KF268948, KX198134, KU955591, and KU955592).

## 5. Conclusions

The current outbreak of ZIKV in Thailand is poorly understood. In this report, we demonstrated the use of molecular techniques for investigating the epidemiology and genetic diversity of ZIKV in mosquitoes collected from ZIKV outbreak areas in Thailand. ZIKV was detected in female, male, and larvae of *Ae. aegypti* and *Cx. quinquefasciatus*, in female and male *Ar. subalbatus*, which might be vectors of ZIKV, and thus causative agents of ZIKV infection in Thailand. This study represents the first detection of ZIKV RNA in *Cx. quinquefasciatus* from Thailand. ZIKV from mosquitoes in Thailand can be divided into two clades that are closely related to those reported in a previous Thai study and another clade related to ZIKV from the Americas. However, extensive surveys and more precise studies of ZIKV infection in mosquitoes covering more areas and larger sample sizes must be performed to understand geographic location and virus interaction and to provide the basis for an effective vector control program. These actions are important keys to prevent the disease from spreading.
